# Diversity of *Manota* Williston (Diptera, Mycetophilidae) in Ulu Temburong National Park, Brunei

**DOI:** 10.3897/zookeys.428.7912

**Published:** 2014-07-24

**Authors:** Jan Ševčík, Heikki Hippa, Rodzay Abdul Wahab

**Affiliations:** 1 University of Ostrava, Faculty of Science, Department of Biology and Ecology & Institute of Environmental Technologies, Chittussiho 10, CZ-710 00 Ostrava, Czech Republic; 2 Gribbylunds allé 2, SE-183 65 Täby, Sweden; 3 Universiti Brunei Darussalam, Institute for Biodiversity and Environmental Research, Tungku Highway, BE1410, Brunei Darussalam

**Keywords:** Insecta, Sciaroidea, Manotinae, fungus gnats, taxonomy, new species, Borneo, Belalong, Tungku, Oriental region

## Abstract

A total of 15 species of *Manota* Williston, 1896 are recorded from Brunei, based on the investigations in 2013-2014. Thirteen species are recorded from Ulu Temburong National Park and three species from the Universiti Brunei Darussalam Campus in Tungku. Six species are described as new to science: *Manota belalongensis* **sp. n.**, *M. kaspraki* **sp. n.**, *M. macrothrix* **sp. n.**, *M. megachaeta* **sp. n.** and *M. pileata* **sp. n.** from Ulu Temburong, and *M. ricina* **sp. n.** from Tungku. New records of the following species are given: *Manota bifida* Hippa & Papp, *M. bruneiensis* Hippa & Ševčík, *M. hyboloma* Hippa & Ševčík, *M. oligochaeta* Hippa, *M. pappi* Hippa, *M. perangulata* Hippa & Ševčík, *M. pollex* Hippa, *M. procera* Hippa and *M. simplex* Hippa.

## Introduction

Fungus gnats (Diptera: Sciaroidea) represent one of the most abundant and diverse groups of insects in forest habitats, in both temperate and tropical regions. Within Sciaroidea, the family Mycetophilidae belongs to the most species rich groups, with some 4500 described species worldwide and possibly the same number of species still awaiting description. The predominantly tropical subfamily Manotinae form a rather uniform and well-defined group that has been proved to be monophyletic in studies based on both morphological (Hippa et al. 2005) and molecular characters (Ševčík et al. 2013). Of the 4 genera included, only *Manota* Williston, 1896 is distributed worldwide with more than 200 described species (cf. Hippa and Kurina 2012, 2013; Hippa and Ševčík 2013) and the number of undescribed species is difficult to estimate because *Manota* is considered as an open-ended taxon (see Bickel 2009). The species inventory of *Manota* in the Oriental Region during the past 10 years has raised the number of species from one (Senior-White 1922) to 89 (for a review of investigations see Hippa and Ševčík 2010, 2013).

**Figure 1.**
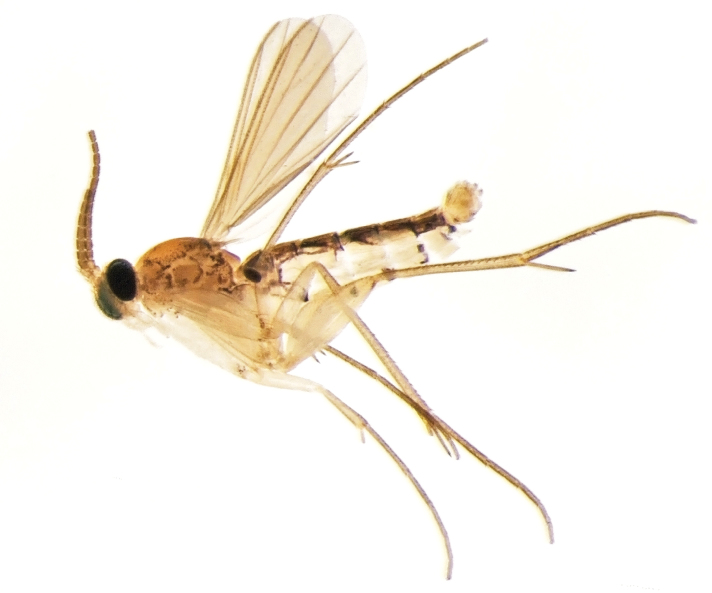
Habitus of *Manota kaspraki* sp. n. (Photo by D. Kaspřák).

The specimens of *Manota* can be easily identifiable among other mycetophilids in collections due to their small size and characteristically reduced wing venation (Fig. 1). On the other hand, the species identification is rather difficult because it is based on the study of male terminalia under a relatively high magnification.

The mycetophilid fauna of Borneo, as well as of the entire Indomalayan Archipelago is still poorly known. Concerning Brunei, only several named species of Mycetophilidae (see Hippa and Ševčík 2010; Papp and Ševčík 2011; Ševčík and Hippa 2010) and four species from the closely related families Diadocidiidae and Keroplatidae (Papp and Ševčík 2005; Ševčík and Papp 2009; Ševčík 2012) have been reported up to the present.

In 2013, collaboration between Universiti Brunei Darussalam and the University of Ostrava was established, resulting in two field trips to Ulu Temburong National Park (February 2013 and January 2014). This contribution is the first from a series of ecological and taxonomic papers devoted to the study of this well preserved and highly diverse lowland rainforest in Brunei. Its aim is to describe 5 new species of *Manota* from Ulu Temburong National Park, to record 9 additional species and discuss the potential species richness in this area. The opportunity is also taken to describe a new species from the university campus near the capital of Brunei, Bandar Seri Begawan, outside the Temburong District.

## Material and methods

**Figure 2.**
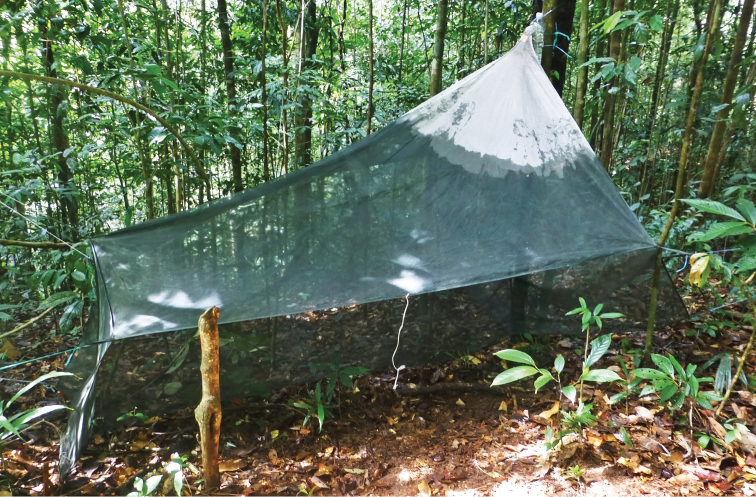
Malaise trap in the rainforest around Kuala Belalong Field Studies Centre (Photo by J. Ševčík).

Most of the material examined was collected with Malaise traps. A total of six Malaise traps (Fig. 2) were operated in the area in 5–17.ii.2013 and 7–18.i.2014. Additional samples were obtained by sweeping the undergrowth of the rainforest with an entomological net.

**Figure 3.**
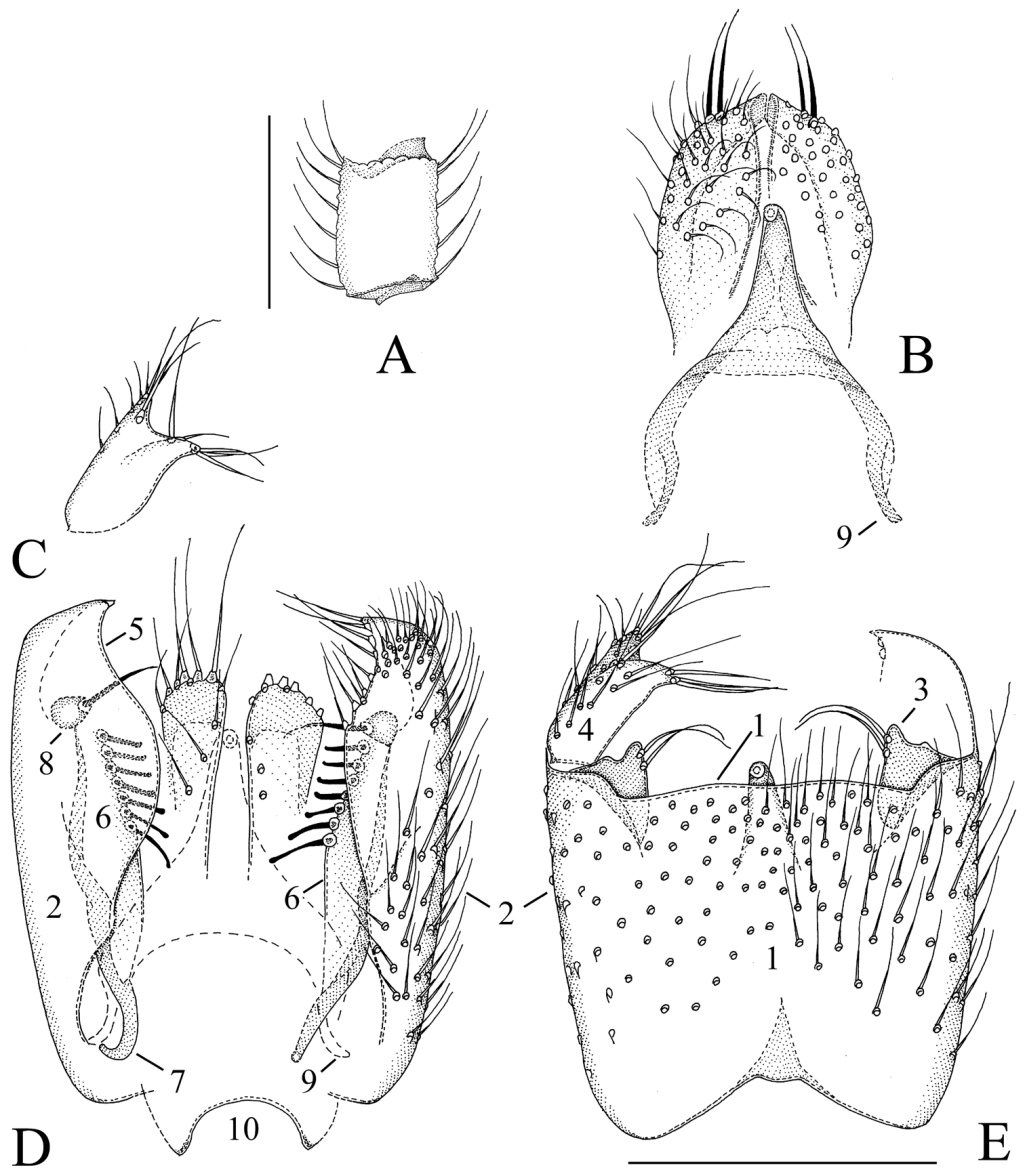
*Manota belalongensis* sp. n. (holotype). **A** Antennal flagellomere 4, lateral view **B** Aedeagus and hypoproct. ventral view **C** Gonostylus, dorsal view **D** Hypopygium, dorsal view **E** Hypopygium, ventral view. Scale 0.1 mm. 1 = sternite 9, 2 = gonocoxa, 3 = parastylar lobe, 4 = gonostylus, 5 = dorsal mesial margin of gonocoxa, 6 = plate-like lobe ventrally from the dorsal mesial margin of gonocoxa, 7 = gonocoxal apodeme, 8 = juxtagonostylar seta, 9 = aedeagal apodeme, 10 = tergite 9.

**Figure 4.**
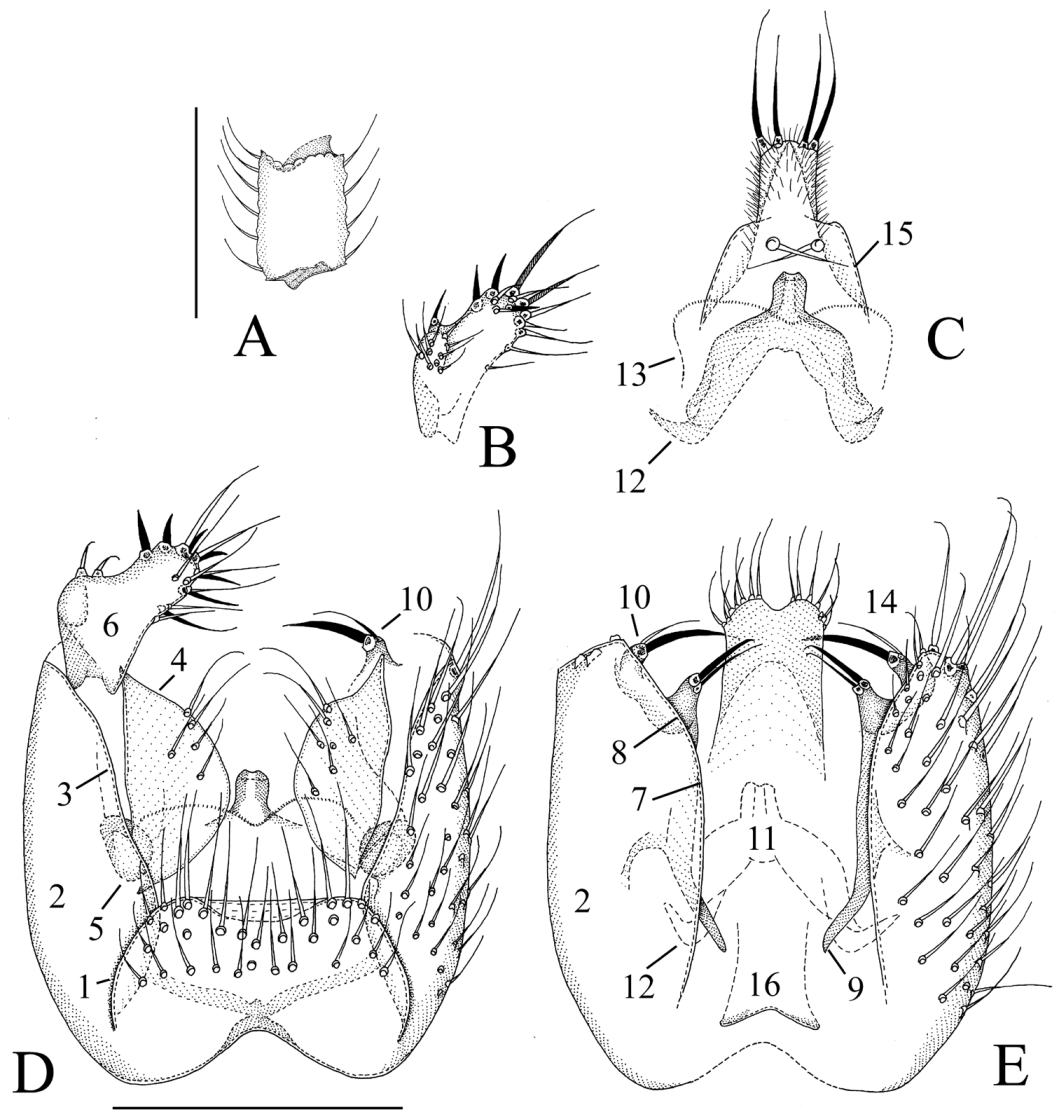
*Manota kaspraki* sp. n. (holotype). **A** Antennal flagellomere 4, lateral view **B** Gonostylus, dorsal view **C** Aedeagus and hypoproct, ventral view **D** Hypopygium, ventral view **E** Hypopygium, dorsal view. Scale 0.1 mm. 1 = sternite 9, 2 = gonocoxa, 3 = ventral mesial margin of gonocoxa, 4 = parastylar lobe, 5 = paraapodemal lobe, 6 = gonostylus, 7 = dorsal mesial margin of gonocoxa, 8 = lobe at dorsal mesial/apical margin of gonocoxa, 9 = gonocoxal apodeme, 10 = juxtagonostylar setae, 11 = aedeagus, 12 = aedeagal apodeme, 13 = membranous lobe, 14 = cerci, 15 = ventral seta of hypoproct (sternite 10), 16 = tergite 9.

The material was collected into and preserved in ethanol. The abdomen or only the apical part of the abdomen was detached from the specimen and macerated in warm concentrated potassium hydroxide (KOH). We also detached the hypopygium beyond segment 8. After washing in water and dehydration in stages of increasing concentrations of alcohol we mounted them in ‘Euparal’ between two pieces of cover slip, which allows the specimen to be studied from both sides under a compound microscope. These preparations are now attached to normal microscope slides by two strips of adhesive tape across their edges and are easily detached when needed. Other parts of the body were not macerated, but after dehydration we mounted the whole flies as they were in ‘Euparal’, lying on their side. The descriptions of the hypopygium should only be taken as rough guidelines to interpret the drawings. The morphological terminology follows our earlier papers (Hippa and Ševčík 2010, 2013). The terminology is also indicated in Figs 3–4. Illustrations were made with the aid of a drawing tube attached to a Leitz Diaplan compound microscope. Wing length is given measured from wing base (and from humeral cross vein) to wing tip.

The material is deposited in the following collections: Universiti Brunei Darussalam, Brunei (UBDC), University of Ostrava, Czech Republic (UOSC), and Natural History Museum, London, Great Britain (BMNH).

### Study area

The study area is located at the Kuala Belalong Field Studies Centre (KBFSC), a research field station of the Universiti Brunei Darussalam. It is situated in the Ulu Temburong National Park; geographic coordinates are 4°33'N, 115°10'E, elevations in the KBFSC surroundings range from 60 (the station) to ca 300 m asl. The topography is very rugged, with narrow ridges and steep slopes. The site represents pristine lowland mixed dipterocarp forest, only slightly touched by human activities (Cranbrook and Edwards 1994; Hédl et al. 2009). The climate is equatorial with average temperatures around 30 °C. There is no dry season and precipitation reaches 4 000 mm. Relative air humidity in the forest exceeds 95% (Dykes 2000). For a review of biological investigations carried out at the station see Cranbrook and Edwards (1994).

## Results

### Descriptions of the new species

#### Manota belalongensis sp. n.

Taxon classificationAnimaliaDipteraMycetophilidae

http://zoobank.org/8BB86E68-1183-41AD-8BFA-3F9BB23437DB

Fig. 3

##### Description.

**Male. Colour.** Head dark brown, face and clypeus paler brown. Antenna brown, scapus and pedicellus paler brown. Mouthparts yellowish. Thorax brown, preepisternum 2 ventrally and episternum 3 paler yellowish. Legs yellowish, apex of coxa 2 and 3, trochanter 3 and basal third of femur 3 infuscated. Wing brownish, halter pale brown with blackish knob. Abdomen brown, sternites paler than tergites. All setosity pale, yellowish or brownish. **Head.** Antennal flagellomere 4, Fig. 3A. Palpomere 3 of maxillary palpus with apico-mesial thumb-like extension, with 4 apically expanded and curved sensilla; palpomere 4 with parasegment; palpomere 5 is 1.5 times as long as palpomere 4. Number of strong postocular setae 10. **Thorax.** Anepisternum setose, with 56 setae; anterior basalare setose, with 8 setae; preepisternum 2 non-setose; laterotergite setose, with 37 setae; episternum 3 setose, with 6 setae. **Legs.** Mid and hind tibial organs absent. **Wing.** R_1_ meeting C well on the basal half of the costal margin; the sclerotized part of M_2_ extending near to the level of the tip of R_1_; wing length 1.4 (1.5) mm. **Hypopygium.** Fig. 3B–E: Sternite 9 laterally fused with gonocoxa, posterior margin extending well over the middle of gonocoxa, near to the base of gonostylus, anterior margin incised, the setae similar to ventral setae of the gonocoxa. Ventral mesial margin of gonocoxa short, simple, the ventral setae of gonocoxa unmodified. Parastylar lobe subtriangular, with 2 setae at postero-mesial margin. Paraapodemal lobe not identifiable. Dorsal mesial margin of gonocoxa simple, posteriorly slightly sigmoid in shape, without megasetae or other stronger setae. Gonocoxa without a postero-lateral lobe. The dorsal setae of gonocoxa unmodified, similar to those on the ventral side. One juxtagonostylar seta present; it is a curved acute megaseta arising from oval basal body which is ca. one third of the length of the seta. Anteriorly from the juxtagonostylar megaseta there is a plate-like lobe bearing a row of 7 blunted rather thin megasetae. Gonostylus small, elongate, apically bilobed, the ventral side setose, dorsal side non-setose, the apical lobes with a few long setae which are not much stronger than the other gonostylar setae. Aedeagus elongate subtriangular, with weak lateral shoulders, the apex curved ventrad. Hypoproct large, extending posteriorly to the apices of gonostyli, number of its ventral setae (sternite 10) ca. 30 on each half, these setae widely distributed over the ventral surface. Cerci mesially separate.

**Female** unknown.

##### Discussion.

*Manota belalongensis* belongs to a large group, world-wide in distribution, in which male sternite 9 is long and laterally fused with the gonocoxa. In the key to Oriental and Palaearctic *Manota* (Hippa 2011) the species runs to *Manota heptacantha* Hippa, 2006 from Peninsular Malaysia and Thailand and is also in most respect very similar to it (cf. Hippa 2006: Fig. 6E, F). It differs by having the gonostylus apically bilobed (simple in *Manota heptacantha*), by having only two setae on the parastylar lobe (several in *Manota heptacantha*) and by having the postero-mesial angle (apex) of the parastylar lobe abruptly narrowed (evenly narrowing in *Manota heptacantha*).

##### Etymology.

The species is named after the river Belalong at which the type locality is situated.

##### Types.

*Holotype*. Male, Brunei, Ulu Temburong N. P., Kuala Belalong Field Studies Centre, 4°32'50"N, 115°09'28"E, 8–18.i.2014, primary lowland rainforest, Malaise trap 4, J. Ševčík & D. Kaspřák leg. (in UBDC).

#### Manota kaspraki sp. n.

Taxon classificationAnimaliaDipteraMycetophilidae

http://zoobank.org/E0F48B63-4F4D-4A59-A0F0-05EAB6CF8286

Figs 1 , 4

##### Description.

**Male. Colour.** Head pale brown, face and clypeus pale yellowish. Antenna brown, scapus and pedicellus paler. Mouthparts yellowish. Thorax pale brown, preepisternum 2 pale yellowish. Legs yellowish. Wing brownish, halter brownish with blackish knob. Abdomen pale brown, sternites paler than tergites. All setosity pale, yellowish or brownish. **Head.** Antennal flagellomere 4, Fig. 4A. Palpomere 3 of maxillary palpus with apicomesial thumb-like extension, with 3 apically expanded and curved sensilla; palpomere 4 with parasegment; palpomere 5 ca. 1.4 times longer than palpomere 4. Number of strong postocular setae 10–11. **Thorax.** Anepisternum setose, with 14–31 setae; anterior basalare setose, with 6–10 setae; preepisternum 2 setose, with 8–11 setae, laterotergite non-setose; episternum 3 setose, with 2–3 setae. **Legs.** Mid and hind tibial organs absent. **Wing.** R_1_ meeting C well on the basal half of the costal margin; the sclerotized part of M_2_ extending near to the level of the tip of R_1_; wing length 1.3–1.5 (1.1–1.4) mm. **Hypopygium.** Fig. 4B–E: Sternite 9 about one third of ventral length of gonocoxa, with sharply delimited convex sides, posterior margin transverse with a wide submembranous notch, anterior margin shallowly incised, the setae similar to adjacent ventral setae of gonocoxa. Ventral mesial margin of gonocoxa simple, sigmoid. Parastylar lobe large, almost semicircular, with 5–7 setae scattered on the ventral surface. Paraapodemal lobe oval, at least partly covered by gonocoxa and parastylar lobe. The dorsal mesial margin of gonocoxa simple, convex, contiguous with the simple oblique posterior margin; posteriorly at the dorsal mesial margin there is a finger-like lobe apically bearing a stronger and a weaker seta; this lobe is very similar to the juxtagonostylar setae in appearance. Two juxtagonostylar setae, one an unmodified acute megaseta, the other a usual seta, both arising from a common basal body which is about one half of the length of the setae. Gonostylus elongate, slightly angled, with sub-basal lateral rounded lobe, the setosity confined to the lateral lobe and the apical part, some of the setae shorter but strong (spine-like megasetae). Aedeagus short, with strong lateral shoulders, the apex straight, not curved ventrad; on the ventral side of aedeagus (Fig. 4C–E) there is a membranous lobe, which may belong to aedeagus. Hypoproct posteriorly extending near to the apex of gonostylus, unusually narrow; ventrally with one seta on each side (sternite 10), postero-dorsally with two strong setae on each side, the microtrichia unusually long. Cerci mesially fused.

**Female** unknown.

##### Discussion.

*Manota kaspraki* is not especially similar to any other described *Manota*. In the key to Oriental and Palaearctic *Manota* it runs to couplet 38 by the following characters: anepisternum setose, preepisternum 2 setose, laterotergite non-setose, anterior basalare setose, gonostylus unilobed and straight (not geniculate). Couplet 38 leads to a large number of species (18). The acute spine-like megasetae apically on the curiously hump-sided gonostylus and the narrow long microtrichose hypoproct distinguish *Manota kaspraki* from any hitherto described *Manota*. The inflated paraapodemal lobe is reminiscent of e.g. *Manota vesicaria* Hippa, 2009 from Thailand, but otherwise the species are not much similar.

##### Etymology.

The species is named after Mr. David Kaspřák, a PhD student at the University of Ostrava, who participated in both the expeditions to Brunei, helped with the installation of Malaise traps and with other field activities.

##### Types.

*Holotype*. Male, Brunei, Ulu Temburong N. P., Kuala Belalong Field Studies Centre, 4°32'50"N, 115°09'28"E, 7–18.i.2014, primary lowland rainforest, Malaise trap 1 (night), D. Kaspřák & J. Ševčík leg. (in UBDC).

*Paratypes*. 2 males with same data as holotype except Malaise trap 1 (ODP) (in UBDC and UOSC); 2 males, the same data except sweeping February 2013 (in BMNH).

#### Manota macrothrix sp. n.

Taxon classificationAnimaliaDipteraMycetophilidae

http://zoobank.org/A5341B31-FF64-44FA-B173-29FD0A157C2B

Fig. 5

##### Description.

**Figure 5.**
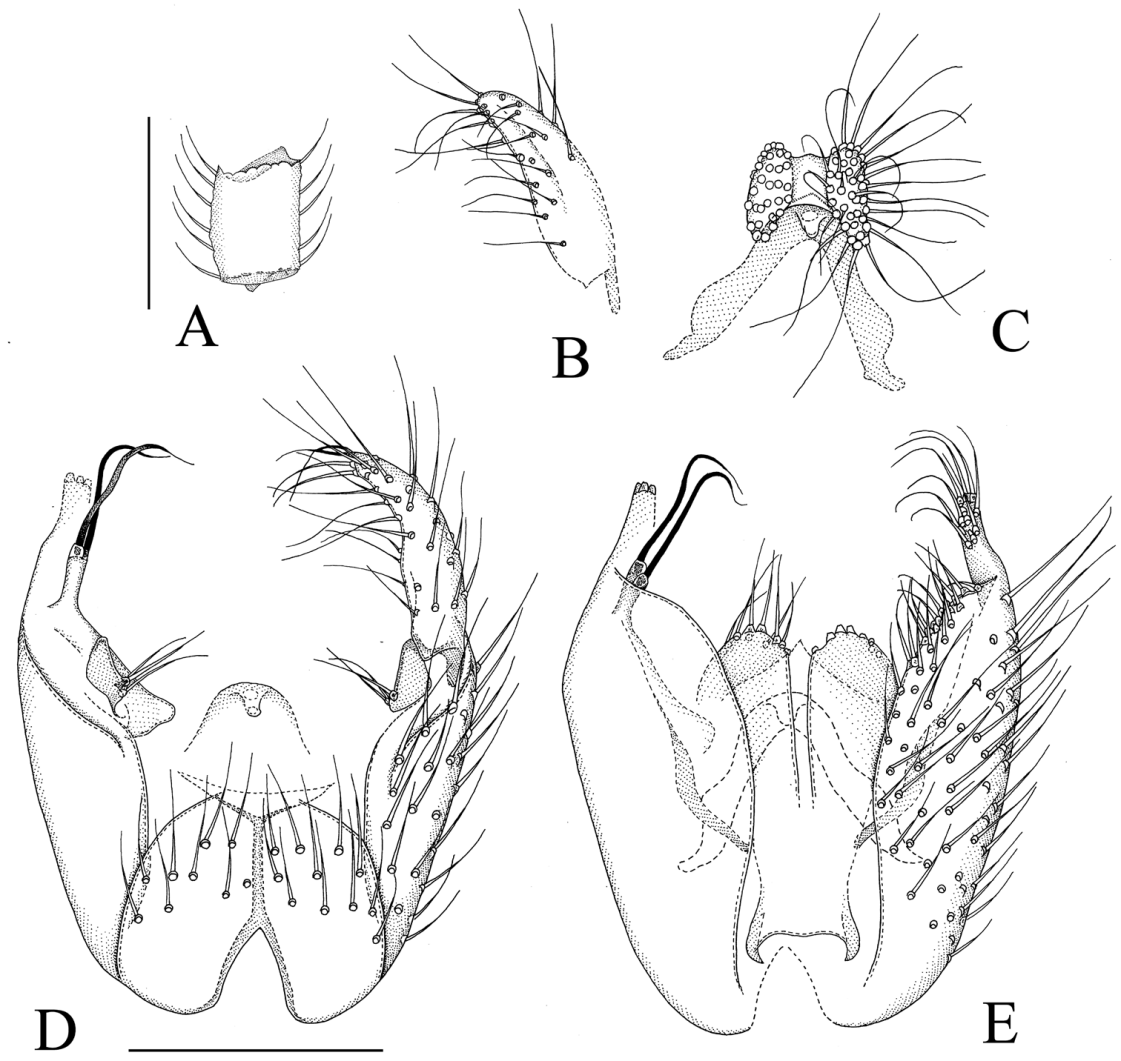
*Manota macrothrix* sp. n. (holotype). **A** Antennal flagellomere 4, lateral view **B** Gonostylus, dorsal view **C** Aedeagus and hypoproct, ventral view **D** Hypopygium, ventral view **E** Hypopygium, dorsal view. Scale 0.1 mm.

**Male. Colour.** Head brown, face and clypeus paler brown. Antenna brown. Mouthparts yellowish. Thorax pale brown, preepisternum 2 ventrally pale yellowish. Legs yellowish, femur 3 with a very slight indication of infuscation. Wing brownish, halter pale brown with blackish knob. Abdomen pale brown, sternites paler than tergites. All setosity pale, yellowish or brownish. **Head.** Antennal flagellomere 4, Fig. 5A. Palpomere 3 of maxillary palpus with apico-mesial thumb-like extension, with 5 apically expanded and curved sensilla; palpomere 4 with parasegment; palpomere 5 is 1.3 times as long as palpomere 4. Number of strong postocular setae 10–11. **Thorax.** Anepisternum setose, with 34–55 setae; anterior basalare non-setose; preepisternum 2 setose, with 15 setae, laterotergite non-setose; episternum 3 setose, with ca. 9 setae. **Legs.** Mid and hind tibial organs absent. **Wing.** R_1_ meeting C well on the basal half of the costal margin; the sclerotized part of M_2_ extending near to the level of the tip of R_1_; wing length 1.4–1.6 (1.5–1.7) mm. **Hypopygium.** Fig. 5B–E: Sternite 9 about one half of ventral length of gonocoxa, with sharply delimited convex sides, posterior margin not well seen in the holotype because the dorsal membranous area extruded, anterior margin deeply incised, the setae similar to adjacent ventral setae of gonocoxa. Ventral mesial margin of gonocoxa simple, sigmoid. Parastylar lobe oblique, subtriangular, with 3 setae antero-mesially (at apex). Paraapodemal lobe well exposed in ventral view. The dorsal mesial margin of gonocoxa simple, convex, posteriorly with a group of tightly placed setae. Gonocoxa posterolaterally with an apically setose apophysis/lobe. Two juxtagonostylar setae, both long apically curved megasetae, both arising from a common basal body, about one third of the length of megasetae. Gonostylus elongate and slightly curved, with moderately long setosity ventrally and dorsally, without megasetae or other setae deviating from the general setosity. Aedeagus short subtriangular, with distinct lateral shoulders, the apex curved ventrad. Hypoproct posteriorly extending to level of middle of gonostylus, the ventral part divided into two elongate oval lobes (sternite 10) covered by ca 30 very long setae on each half, postero-dorsally with a few both fine and strong setae. Cerci mesially separate.

**Female** unknown.

##### Discussion.

*Manota macrothrix* belongs to a large group of species, common in the Oriental region, all of which have a well-developed apico-mesial thumb-like extension on palpomere 3, a setose anepisternum and preepisternum 2, a non-setose anterior basalare and laterotergite, a short vein R_1_, laterally free tergite 9, an oblique sickle-shaped or subtriangular parastylar lobe, well developed paraapodemal lobe, an apically setose apophysis at the apico-dorsal margin of the gonocoxa, two juxtagonostylar megasetae arising from a common basal body and a rather unmodified elongate gonostylus. Within this group *Manota macrothrix* is similar to *Manota dolichothrix* Hippa & Ševčík, 2010 from Sabah by having extremely long setae ventrally on the hypoproct (on sternite 10) but differs in having these setae widely scattered on each half of the hypoproct, while they are in a single row on each half in *Manota dolichothrix*. Further, in *Manota macrothrix* the subapical setae of gonostylus are shorter, less than twice longer than the medial width of gonostylus, while in *Manota dolichothrix* they are almost three times as long as the width of gonostylus. There are many minor differences between the two species (Fig. 5B–E, Hippa and Ševčík 2010: Fig. 6B–C).

##### Etymology.

The name is a Latinized Greek noun, *macrothrix*, long-hair, referring to the very long setae on the hypoproct.

##### Types.

*Holotype*. Male, Brunei, Ulu Temburong N. P., Kuala Belalong Field Studies Centre, 4°32'50"N, 115°09'28"E, 7–17.i.2014, primary lowland rainforest, Malaise trap 2, J. Ševčík & D. Kaspřák leg. (in UBDC).

*Paratype*. 1 male, Brunei, Ulu Temburong, 14.ii – 9.iii 1982, Malaise trap, M. C. Day leg. (in BMNH).

#### Manota megachaeta sp. n.

Taxon classificationAnimaliaDipteraMycetophilidae

http://zoobank.org/661EA393-5728-4952-84BC-BCBCB50061CB

Fig. 6

##### Description.

**Figure 6.**
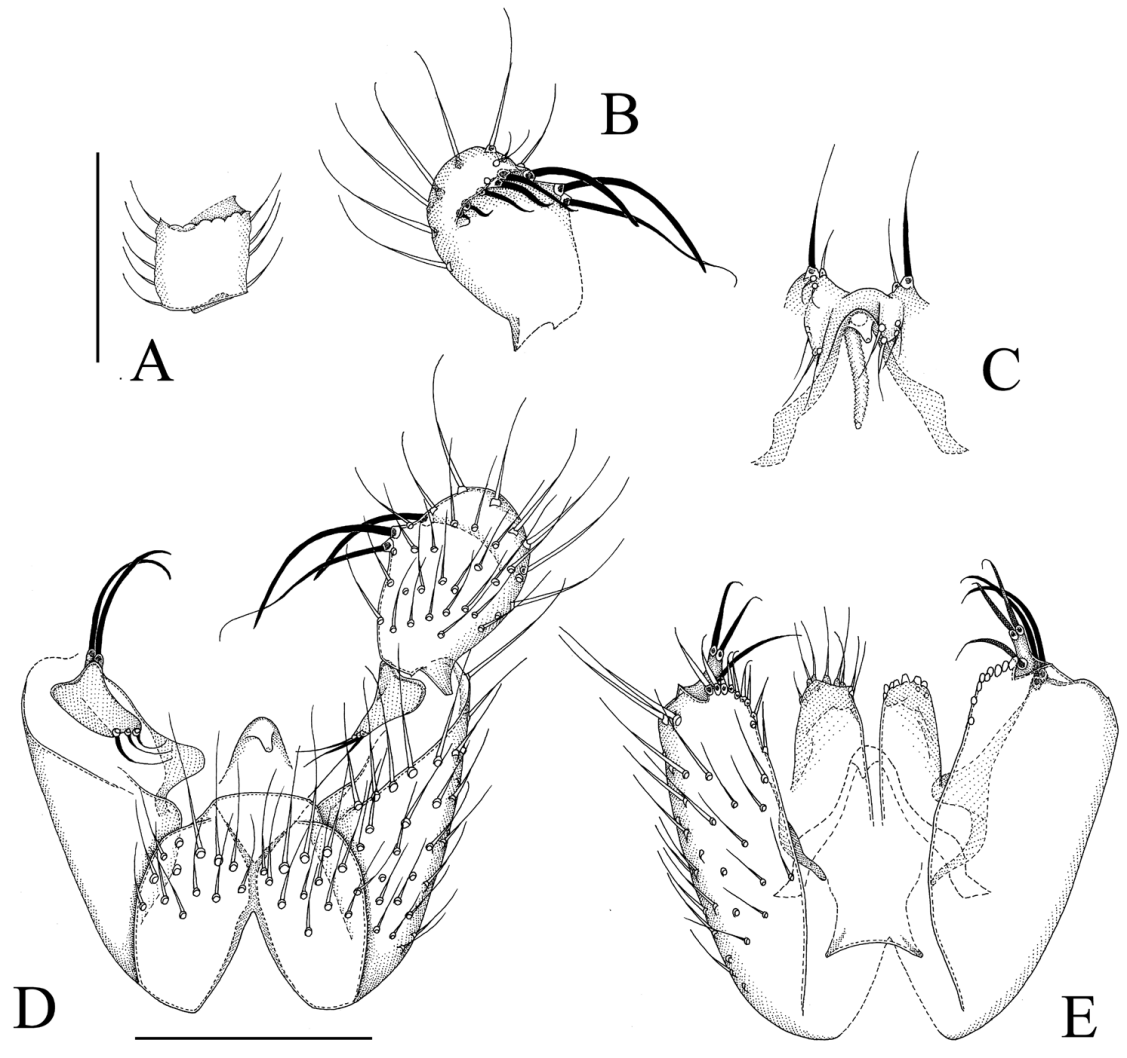
*Manota megachaeta* sp. n. (holotype). **A** Antennal flagellomere 4, lateral view **B** Gonostylus, dorsal view **C** Aedeagus and hypoproct, ventral view **D** Hypopygium, ventral view **E** Hypopygium, dorsal view. Scale 0.1 mm.

**Male. Colour.** Head brown, face and clypeus paler brown. Antenna brown, scapus and pedicellus paler brown. Mouthparts yellowish. Thorax pale brown, preepisternum 2 ventrally paler yellowish. Legs yellowish. Wing yellowish brown, halter pale brown with blackish knob. Abdomen pale brown, sternites paler than tergites. All setosity pale, yellowish or brownish. **Head.** Antennal flagellomere 4, Fig. 6A. Palpomere 3 of maxillary palpus with apicomesial thumb-like extension, with 5 apically expanded and curved sensilla; palpomere 4 with parasegment; palpomere 5 is 1.3 times as long as palpomere 4. Number of strong postocular setae ca 9. **Thorax.** Anepisternum setose, with 33 setae; anterior basalare non-setose; preepisternum 2 setose, with 14 setae, laterotergite non-setose; episternum 3 setose, with ca. 11 setae. **Legs.** Mid and hind tibial organs absent. **Wing.** R_1_ meeting C well on the basal half of the costal margin; the sclerotized part of M_2_ extending near to the level of the tip of R_1_; wing length 1.4 (1.5) mm. **Hypopygium.** Fig. 6B–E: Sternite 9 about one half of ventral length of gonocoxa, with sharply delimited convex sides, posterior margin convex, anterior margin deeply incised, the setae posteriorly stronger than anteriorly. Ventral mesial margin of gonocoxa simple, concave. Parastylar lobe oblique, subtriangular, with 3 setae anteriorly (at apex). Paraapodemal lobe well exposed in ventral view. The dorsal mesial margin of gonocoxa simple, convex, postero-mesially with a weak setose lobe; posterior margin simple, at posterior margin on a more ventral level with a finger-like lobe bearing two apical and one sub-basal acute curved megasetae. Generally the dorsal setae of gonocoxa similar to the ventral ones. Two juxtagonostylar setae, both long curved acute megasetae arising from a common low inconspicuous basal body. Gonostylus oval, with moderately long setosity ventrally, the dorsal side non-setose except for a transverse row of megasetae on apical half, the megasetae acute and increasing in length towards the mesial margin. Aedeagus short subtriangular, with distinct lateral shoulders, the apex curved ventrad; in Fig. 6C the medially visible part is vas deferens. Hypoproct posteriorly extending to level of base of gonostylus, the ventral part divided into two elongate lobes (sternite 10) with 4 setae each, postero-dorsally with a few both fine and strong setae. Cerci mesially separate.

**Female** unknown.

##### Discussion.

By the following characters *Manota megachaeta* runs to couplet 65 in the key to Oriental and Palaearctic *Manota* (Hippa 2011): anepisternum setose, preepisternum 2 setose, laterotergite non-setose, anterior basalare non-setose, juxtagonostylar setae/megasetae simple and not greatly expanded, the dorsal posterior margin of gonocoxa with a cylindrical, not flat, setigerous lobe. This couplet leads to *Manota anceps* Hippa & Ševčík, 2010, *Manota duplex* Hippa, 2006, *Manota perpusilla* Hippa, 2006, *Manota vesicaria* Hippa, 2009, *Manota pellii* Hippa, 2008 and *Manota hexacatha* Hippa & Ševčík, 2010. However, *Manota megachaeta* is not especially similar to any of them and is easily distinguished by the subapical transverse row of unusually strong megasetae dorsally on the gonostylus.

##### Etymology.

The name is a Latinized Greek noun, *megachaeta*, large seta, referring to the unusually long megasetae on the gonostylus.

##### Types.

*Holotype*. Male, Brunei, Ulu Temburong N. P., Kuala Belalong Field Studies Centre, 4°32'50"N, 115°09'28"E, 7–18.i.2014, primary lowland rainforest, Malaise trap 1 (DOP), J. Ševčík leg. (in UBDC).

#### Manota pileata sp. n.

Taxon classificationAnimaliaDipteraMycetophilidae

http://zoobank.org/B00859B6-7712-4083-9297-E617CBABA8F7

Fig. 7

##### Description.

**Figure 7.**
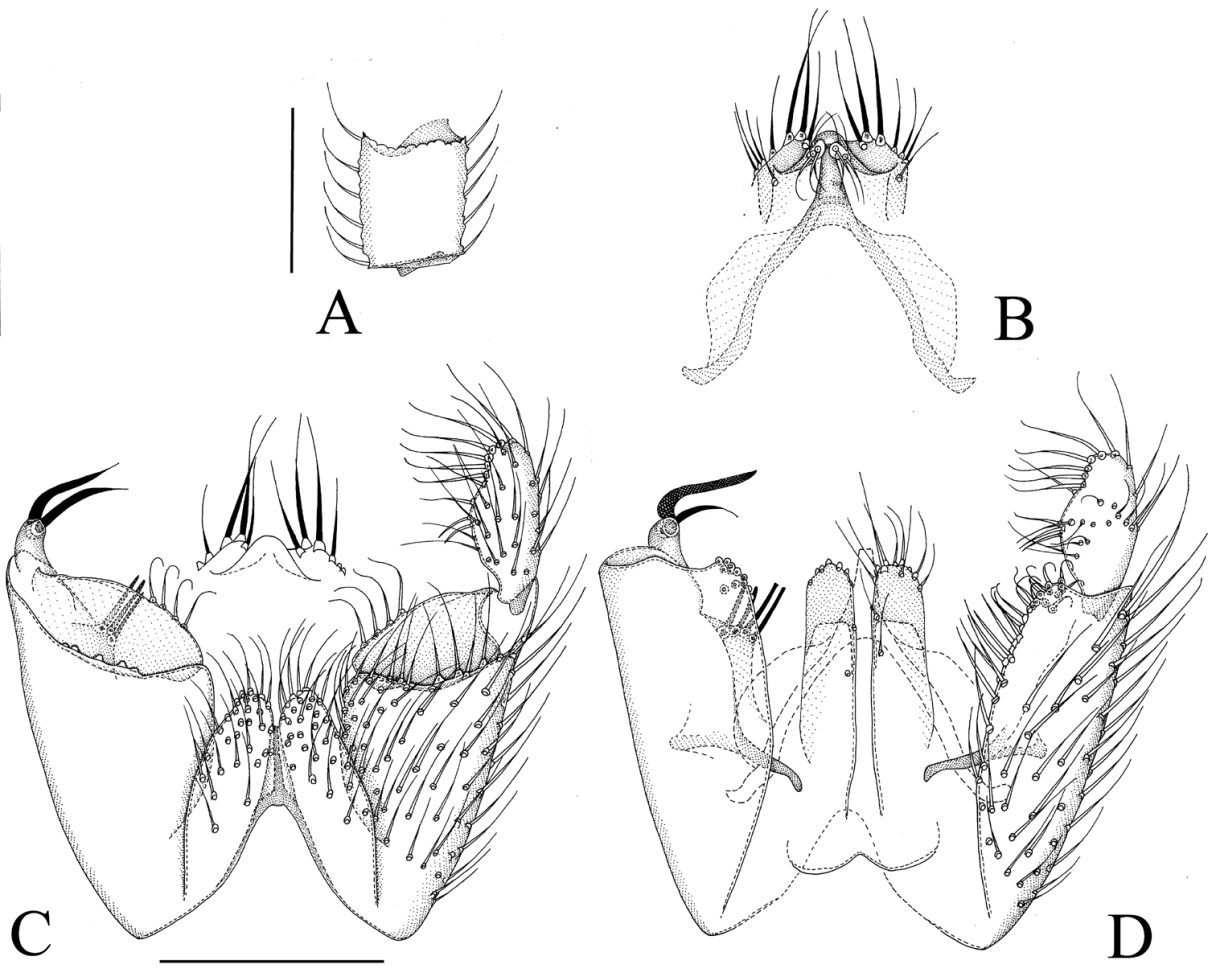
*Manota pileata* sp. n. **A** Antennal flagellomere 4, lateral view **B** Aedeagus and hypoproct, ventral view **C** Hypopygium, ventral view **D** Hypopygium, dorsal view. Scale 0.1 mm.

**Male. Colour.** Head brown, face and clypeus paler brown. Antenna brown. Mouthparts yellowish. Thorax brown, preepisternum 2 and episternum 3 ventrally paler yellowish. Legs yellowish, apical fourth of femur 3 infuscated. Wing brownish, halter pale brownish with blackish knob. Abdomen brown, sternites paler than tergites. All setosity pale, yellowish or brownish. **Head.** Antennal flagellomere 4, Fig. 7A. Palpomere 3 of maxillary palpus with apico-mesial thumb-like extension, with 4 apically expanded and curved sensilla; palpomere 4 with parasegment; palpomere 5 is 1.7 times as long as palpomere 4. Number of strong postocular setae 11. **Thorax.** Anepisternum setose, with 33 setae; anterior basalare setose, with 12 setae; preepisternum 2 setose, with ca. 17 setae, laterotergite non-setose; episternum 3 setose, with ca. 12 setae. **Legs.** Mid tibial organ absent; hind tibial organ present. **Wing.** R_1_ meeting C well on the basal half of the costal margin; the sclerotized part of M_2_ extending near to the level of the tip of R_1_; wing length 1.5 (1.6) mm. **Hypopygium.** Fig. 7B–D: Sternite 9 about one half of ventral length of gonocoxa, with sharply delimited convex sides, posterior margin with narrow deep cleft, anterior margin deeply incised, the setae similar to adjacent ventral setae of gonocoxa. Ventral mesial margin of gonocoxa angled. Parastylar lobe large, elongate oval, oblique, with 4 setae at mesial margin. Paraapodemal lobe not identified; at the place where a paraapodemal lobe is usually visible there is a plate-like lobe with three megasetae. The dorsal mesial margin of gonocoxa simple, convex, posteriorly forming a weak lobe with marginal and ventral setae; posterior margin of gonocoxa transverse, simple. The dorsal setae of gonocoxa similar to ventral ones. Two juxtagonostylar setae, both long curved megasetae, the dorsal one stronger than the ventral one, both arising from a common basal body, about one half of the length of the stronger megaseta. Gonostylus elongate oval, with moderately long setosity ventrally and dorsally, fewer and partly weaker setae ventrally, the apico-mesial setae longer than the others, at the middle of mesial margin a few setae which are thick, rather short and which differ from the other setosity. Aedeagus short subtriangular, with distinct lateral shoulders, the apex curved ventrad. Hypoproct posteriorly extending to level of middle of gonostylus, the ventral part (sternite 10) posteriorly with non-setose lobe which have a pair of small oval processes anteriorly bearing three setae each. These processes partly surrounding the apex of aedeagus. Postero-dorsal part of hypoproct with a few both fine and strong setae. Cerci mesially separate.

**Female** unknown.

##### Discussion.

In the key to Oriental and Palaearctic *Manota*, *Manota pileata* runs through couplet 45 to couplet 50 including the Eastern-Palaearctic *Manota indahae* Hippa & Kjaerandsen, 2010 by the following characters: anepisternum setose, preepisternum 2 setose, laterotergite non-setose, gonostylus one-lobed, parastylar lobe present and cerci medially separate, gonostylus without blunt-ended megasetae on apical half, gonostylus without comb-like row of five setae subbasally at the ventral mesial margin, aedeagus apically narrow, without ear-like apico-lateral lobes, parastylar lobe in anterior–posterior direction short, at most twice as long as broad, aedeagus apically symmetrical, the setae medio-dorsally on gonostylus fine, not deviating from the other gonostylar setosity and the dorsal mesial margin of gonocoxa without a thumb-like lobe posteriorly. The two species are not very similar: *Manota pileata* is distinguished from *Manota indahae* e.g. by the following characters: parastylar lobe has only 4 setae (numerous in *Manota indahae*), dorsally from the parastylar lobe there is a plate-like lobe bearing three megasetae (no such lobe), medio-ventrally on the hypoproct there is a rounded lobe (no lobe), and posterior margin of sternite 9 with a cleft (without). Even if the outline of the posterior part of aedeagus is symmetrical it is seen that there is some asymmetry inside (Fig. 7B).

##### Etymology.

The name is a Latin adjective, *pileatus*, -*a*, -*um*, capped, referring to the cap- or hood-like lobes enclosing the apex of aedeagus.

##### Types.

*Holotype*. Male, Brunei, Ulu Temburong N. P., Kuala Belalong Field Studies Centre, 4°32'50"N, 115°09'28"E, 7–17.i.2014, primary lowland rainforest, Malaise trap 2, Ševčík & Kaspřák leg. (in UBDC).

#### Manota ricina sp. n.

Taxon classificationAnimaliaDipteraMycetophilidae

http://zoobank.org/D4232D67-2085-47D1-895B-E25C95CCB2EB

Fig. 8

##### Description.

**Figure 8.**
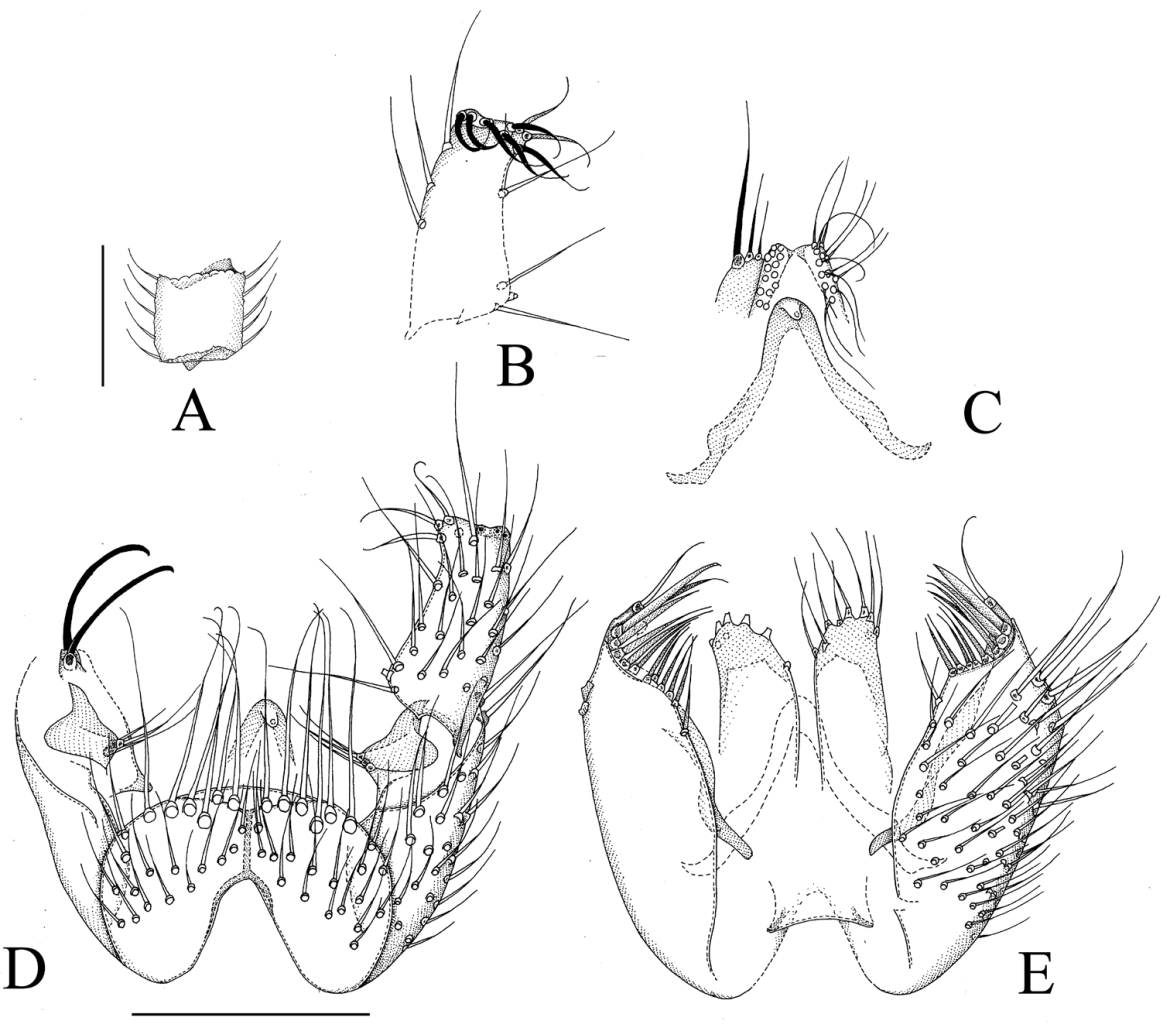
*Manota ricina* sp. n. (holotype). **A** Antennal flagellomere 4, lateral view. **B** Gonostylus, dorsal view **C** Aedeagus and hypoproct, ventral view **D** Hypopygium, ventral view **E** Hypopygium, dorsal view. Scale 0.1 mm.

**Male. Colour.** Head brown, face and clypeus paler brown. Antenna brown. Mouthparts yellowish. Thorax brown, preepisternum 2 pale yellowish. Legs yellowish. Wing brownish, halter brownish with blackish knob. Abdomen brown, sternites paler than tergites. All setosity pale, yellowish or brownish. **Head.** Antennal flagellomere 4, Fig. 8A. Palpomere 3 of maxillary palpus with apico-mesial thumb-like extension, with 5 apically expanded and curved sensilla; palpomere 4 with parasegment; palpomere 5 is 1.4 times as long as palpomere 4. Number of strong postocular setae 9. **Thorax.** Anepisternum setose, with 50 setae; anterior basalare non-setose; preepisternum 2 setose, with 30 setae, laterotergite non-setose; episternum 3 setose, with ca. 18 setae. **Legs.** Mid and hind tibial organs absent. **Wing.** R_1_ meeting C well on the basal half of the costal margin; the sclerotized part of M_2_ extending near to the level of the tip of R_1_; wing length 1.5 (1.6) mm. **Hypopygium.** Fig. 8B–E: Sternite 9 about half of ventral length of gonocoxa, with sharply delimited convex sides which are contiguous with the convex posterior margin, anterior margin deeply incised, the setae at posterior margin very long and strong, on other parts similar to adjacent ventral setae of gonocoxa. Ventral mesial margin of gonocoxa simple, concave. Parastylar lobe oblique, subtriangular, with 3 setae antero-mesially (at apex). Paraapodemal lobe well exposed in ventral view. The dorsal mesial margin of gonocoxa simple, sigmoid, posteriorly with a row of prominent closely placed setae which become broader and flattened towards the posterior end of the row; posteriorly from the row of setae there is a short finger-like lobe with one apical seta. The general dorsal setosity of gonocoxa similar to the ventral one. Two juxtagonostylar setae present, both long curved megasetae arising from a short common basal body, about one fifth of the length of megasetae. Gonostylus elongate subquadrangular, with moderately long setosity ventrally, dorsally non-setose except for a few very strong setae at apical margin on a slightly tuberculate area. Aedeagus subtriangular, with small lateral shoulders, the apex curved ventrad. Hypoproct posteriorly extending to level of basal third of gonostylus, the ventral part with a pair of elongate lobes (sternite 10) bearing ca. 15 scattered setae each, the posterodorsal part with one strong and a couple of weaker setae on each half. Cerci mesially separate.

**Female** unknown.

##### Discussion.

*Manota ricina* resembles *Manota curvata* Hippa, 2006 from Peninsular Malaysia and Sumatra in many respects and follows it in the key to Oriental and Palaearctic *Manota* to couplet 70 by the following characters: anepisternum setose, preepisternum 2 setose, laterotergite non-setose, juxtagonostylar megasetae simple, not expanded, gonocoxa with a conspicuous apicodorsal lobe which is fully exposed, this lobe is flattened, not cylindrical. *Manota ricina* is similar to *Manota curvata* by having the posteriormost setae at the margin of the above-mentioned lobe flat, blade-like, but the shape of the gonostylus is different: in *Manota ricina* it is about twice longer than broad, rather straight, in *Manota curvata* it is more than 4 times longer than broad and curved. We suspect that the small finger-like lobe posteriormost in the row of setae at the mesial margin of the gonocoxa is actually the lobe mentioned above and is not flattened and in this respect the key possibly needs to be revised. *Manota ricina* also differs from *Manota curvata* by the chaetotaxy of the gonostylus, the strong apicodorsal setae being lacking in the latter.

##### Etymology.

The name is a Latin adjective, *ricinus, -a, -um*, veiled, referring to the veil-like fringe of setae posteriorly on the sternite 9.

##### Types.

*Holotype*. Male, Brunei, Tungku, UBD Campus, nr KBFSC Headquarters, 4°58'35"N, 114°53'26"E, 19–22.i.2014, secondary forest, Malaise trap, J. Ševčík & D. Kaspřák leg. (in UBDC).

### Records of other *Manota* species from Brunei

#### Manota bifida

Taxon classificationAnimaliaDipteraMycetophilidae

Hippa & Papp, 2007

##### Material studied.

10 males, Brunei, Ulu Temburong N. P., Kuala Belalong Field Studies Centre, 5–17.ii.2013, primary lowland rainforest, Malaise trap 3b, J. Ševčík leg. (5 in UBDC, 5 in UOSC); 5 males with the same data except 7–17.i.2014, Malaise trap 2, J. Ševčík & D. Kaspřák leg. (3 in UBDC, 2 in UOSC); 1 male with the same data except 7–18.i.2014, Malaise traps in gaps, I. H. Tuf leg. (in UOSC).

##### Remarks.

The species was earlier known from Thailand (Hippa and Papp 2007) and Brunei (Hippa and Ševčík 2010).

#### Manota bruneiensis

Taxon classificationAnimaliaDipteraMycetophilidae

Hippa & Ševčík, 2010

##### Material studied.

1 male, Brunei, Ulu Temburong N. P., Kuala Belalong Field Studies Centre, 5–17.ii.2013, primary lowland rainforest, Malaise trap 1, J. Ševčík leg. (in UBDC); 1 male with the same data except 7–17.i.2014, Malaise trap 2, J. Ševčík & D. Kaspřák leg. (in UOSC).

##### Remarks.

The species was earlier known only by the type material from Brunei (Hippa and Ševčík 2010).

#### Manota hyboloma

Taxon classificationAnimaliaDipteraMycetophilidae

Hippa & Ševčík, 2010

##### Material studied.

1 male, Brunei, Ulu Temburong N. P., Kuala Belalong Field Studies Centre, primary lowland rainforest, sweeping, February 2013, J. Ševčík leg. (in UBDC).

##### Remarks.

The species was earlier known only by the type material from Brunei (Hippa and Ševčík 2010).

#### Manota oligochaeta

Taxon classificationAnimaliaDipteraMycetophilidae

Hippa, 2006

##### Material studied.

1 male, Brunei, Ulu Temburong N. P., Kuala Belalong Field Studies Centre, 7–17.i.2014, primary lowland rainforest, Malaise trap 2, J. Ševčík & D. Kaspřák leg. (in UOSC); 1 male, Brunei, Tungku, UBD Campus, nr KBFSC Headquarters, 19–22.i.2014, secondary forest, Malaise trap, J. Ševčík & D. Kaspřák leg. (in UBDC).

##### Remarks.

The species was earlier known from Peninsular Malaysia (Hippa 2006, 2008) and Thailand (Hippa 2009, 2011; Hippa and Papp 2007).

#### Manota pappi

Taxon classificationAnimaliaDipteraMycetophilidae

Hippa, 2006

##### Material studied.

1 male, Brunei, Ulu Temburong N. P., Kuala Belalong Field Studies Centre, 7–17.i.2014, primary lowland rainforest, Malaise trap 3, J. Ševčík & D. Kaspřák leg. (in UBDC); 1 male with the same data except 8–18.i.2014, Malaise trap 4 (in UOSC); 1 male with the same data except 15.ii.2013, sweeping (in UOSC).

##### Remarks.

The species was earlier known from Peninsular Malaysia (Hippa 2006, 2008), Brunei and Malaysia, Sabah (Hippa and Ševčík 2010).

#### Manota perangulata

Taxon classificationAnimaliaDipteraMycetophilidae

Hippa & Ševčík, 2010

##### Material studied.

1 male, Brunei, Ulu Temburong N. P., Kuala Belalong Field Studies Centre, 7–18.i.2014, primary lowland rainforest, Malaise trap 1 (night), J. Ševčík leg. (in UBDC); 1 male with the same data except 8–18.i.2014, Malaise trap 4, J. Ševčík & D. Kaspřák leg. (in UOSC).

##### Remarks.

The species was earlier known from Brunei (Hippa and Ševčík 2010) and Thailand (Hippa 2011).

#### Manota pollex

Taxon classificationAnimaliaDipteraMycetophilidae

Hippa, 2006

##### Material studied.

1 male, Brunei, Ulu Temburong N. P., Kuala Belalong Field Studies Centre, 5–17.ii.2013, primary lowland rainforest, Malaise trap 3b, J. Ševčík leg. (in UBDC).

##### Remarks.

The species was earlier known from Peninsular Malaysia (Hippa 2006, 2008) and Thailand (Hippa 2011).

#### Manota procera

Taxon classificationAnimaliaDipteraMycetophilidae

Hippa, 2006

##### Material studied.

1 male, Brunei, Bandar Seri Begawan, UBD Campus, nr KBFSC Headquarters, 19–22.i.2014, secondary forest, Malaise trap, J. Ševčík & D. Kaspřák leg. (in UBDC).

##### Remarks.

The species was earlier known from Peninsular Malaysia (Hippa 2006, 2008) and Thailand (Hippa 2009; Hippa and Papp 2007).

#### Manota simplex

Taxon classificationAnimaliaDipteraMycetophilidae

Hippa, 2006

##### Material studied.

1 male, Brunei, Ulu Temburong N. P., Kuala Belalong Field Studies Centre, primary lowland rainforest, sweeping, January 2013, J. Ševčík leg. (in UBDC); 1 male with the same data except 7–17.i.2014, Malaise trap 2, J. Ševčík & D. Kaspřák leg. (in UOSC); 1 male with the same data except Malaise trap 2 (in UBDC).

##### Remarks.

The species was earlier known from Peninsular Malaysia (Hippa 2006, 2008), Thailand (Hippa 2008, 2009, 2011) and Malaysia, Sabah (Hippa and Ševčík 2010).

## Discussion

### Species richness of *Manota* in Brunei

A total of 13 species of *Manota* is reported here from the relatively small area in Ulu Temburong National Park. Two additional species are known from the coastal area of the Universiti Brunei Darussalam campus in Tungku. One species (*Manota oligochaeta*) is common to both these areas. Out of the total of 15 species, 6 were already reported from Brunei by Hippa and Ševčík (2010). These numbers are based on a relatively limited sampling during two field trips to the area and they will definitely be increasing with future studies. Additional four species of *Manota*, not collected in 2013 and 2014, were reported from Ulu Temburong by Hippa and Ševčík (2010), so the total number of *Manota* species known from Ulu Temburong National Park is 17 and that for the entire Brunei is 19.

If we use the Chao 1 formula (Chao 1984; Colwell and Coddington 1994) to calculate the estimated true species diversity of *Manota* in Ulu Temburong National Park, the resulting number would be 29. For comparison, in a rainforest in Peninsular Malaysia (Ulu Gombak Field Study Centre), a total of 27 sympatric species were recorded (Hippa 2006) and the potential species richness could be estimated as 35.

Altogether, more than 50 species of Mycetophilidae and 16 species of Keroplatidae have so far been collected in Ulu Temburong National Park. The patterns of diversity of these taxocoenoses will be treated in a separate paper (Ševčík et al., in prep.).

### Notes on diurnal activity

One trap was emptied 3 times a day (at 7 am, 1 pm and 7 pm), so that it was possible to acquire also data on diurnal and night activity of several species. This will be subject of a separate paper (Kaspřák et al., in prep.) but concerning *Manota*, *Manota kaspraki* and *Manota perangulata* were collected during the night, *Manota bruneiensis* and *Manota megachaeta* in the morning, and *Manota bruneiensis* in the afternoon, indicating a day-long activity of *Manota* species in the tropics. These are the first records of diurnal activity of *Manota* species.

## Supplementary Material

XML Treatment for Manota belalongensis

XML Treatment for Manota kaspraki

XML Treatment for Manota macrothrix

XML Treatment for Manota megachaeta

XML Treatment for Manota pileata

XML Treatment for Manota ricina

XML Treatment for Manota bifida

XML Treatment for Manota bruneiensis

XML Treatment for Manota hyboloma

XML Treatment for Manota oligochaeta

XML Treatment for Manota pappi

XML Treatment for Manota perangulata

XML Treatment for Manota pollex

XML Treatment for Manota procera

XML Treatment for Manota simplex
